# Patient and family trajectories of mitochondrial disease: diversity, uncertainty and genetic risk

**DOI:** 10.1186/2195-7819-9-2

**Published:** 2013-04-15

**Authors:** Rebecca Dimond

**Affiliations:** School of Social Sciences, Cardiff University, Glamorgan Building, King Edward VII Avenue, Cardiff, CF10 3WT UK

## Abstract

Mitochondrial disease can be a devastating, degenerative illness, with limited treatment and no cure. Novel reproductive techniques involving mitochondria donation present an opportunity for women with mitochondrial disease to prevent the transmission of disease to her offspring. Current IVF techniques, such as pre-implantation genetic diagnosis, reduce but do not eliminate the risk for the child. However, knowledge of the contexts within which this disease is experienced and reproductive decisions are made is limited. This article draws on qualitative interviews with adult patients to explore the practical realities of living with mitochondrial disease. Three key themes were identified; the personal and familial experiences of illness, age and generation as factors in shaping patient experience and the importance of experiential knowledge in making sense of reproductive choice. Overall, this article identifies potential barriers to patients accessing reproductive technologies highlighting how the complex nature and uncertain trajectory of mitochondrial disease poses considerable challenges for patients, practitioners and policy makers.

## Introduction

This research has taken place within an evolving landscape of genetic medicine. IVF techniques preventing the transmission of mitochondrial disease from mother to child provide a technological solution to a disease with no cure and extremely limited treatment. The process involves removing the nucleus of an egg with faulty mitochondria, and placing it into a donated, enucleated egg containing healthy mitochondria. As mitochondria contain DNA, these ‘germ line’ therapies are currently prohibited and a change in law would be necessary before they can be offered to patients. The techniques have attracted intense interest and speculation (Bredenoord & Braude 2010; Sample 2012). The Human Fertilisation and Embryology Authority (HFEA) have launched a consultation seeking the views of the public and stakeholders, the results of which will be published in 2013.

Following a call for evidence from patients, professionals and publics, the Nuffield Council on Bioethics recommended the provision of information and support to patients when negotiating novel reproductive techniques (Nuffield Council on Bioethics 2012). In contrast to the extensive interest in the ethical challenges presented by mitochondrial donation, the impact of the disease on patients and families, particularly the adult experience of disease, has attracted much less attention and reflection. This lack of understanding of the adult experience is critical as it is adult women with mitochondrial disease who are the target population for reproductive techniques yet the needs of these patients have yet to be articulated.

Mitochondria are small structures contained in the cytoplasm of a cell, producing energy in the form of ATP (adenosine triphosphate). Each cell contains hundreds to thousands of mitochondria, depending on the energy requirements of particular tissues. The term ‘mitochondrial disease’ was introduced in the late 1980’s to describe the impact of mitochondria dysfunction, which can be due to mutations in either nuclear or mitochondrial DNA sequences. As mitochondria are derived through the oocyte (only one case of paternal inheritance of mitochondria DNA has been identified- Schwartz & Vissing 2002), disease caused by mutations of mitochondrial DNA display a maternal inheritance pattern. Both sexes can inherit the disease but it is only women who are at risk of transmitting the disease to their children. The impact of mitochondrial disease is extremely variable, depending on which organs are affected and to what extent. Symptoms can include extreme fatigue, cardiomyopathy, diabetes, difficulties with mobility and balance, digestive disorders, deafness, epilepsy and restricted sight. Mitochondrial disease is an umbrella label which encompasses a range of disorders. The distinctions in classification and implications for clinical management are beyond the scope of this paper and are described elsewhere (DiMauro 2011; Kisler et al. 2010). An expansive range of symptoms and levels of severity, a complex relationship between genotype and phenotype and the potential for changes in mutation ratio over time presents considerable difficulties for ascertaining prevalence of disease within the population (Schaefer et al. 2008) and for diagnosis, clinical management and genetic counselling (Brown et al. 2006; Poulton et al. 2010; Bredenoord et al. 2009).

Accounts of mitochondrial donation, in media coverage in particular, forecast that if these techniques are made available then this will ‘halt’, ‘eliminate’ or ‘eradicate’ mitochondrial disease from families (see, for example Ahuja 2012). The central tenant of this article is to question this assumption of technological determinism which is in direct contrast to the findings of this study and the wealth of sociological research highlighting the diverse contexts within which the boundaries of health, illness, and risk are negotiated. Three key themes are explored: the personal and familial experiences of illness, age and generation as factors in shaping patient experience and the importance of experiential knowledge in making sense of reproductive choice. This is the first empirical study to examine how the complexity and uncertainty of mitochondrial disease impacts on the patient experience and experiences of reproductive risk, providing a unique contribution to an under researched yet highly topical area.

This article initiates discussion on the patient experience of mitochondrial disease. It is based on the accounts of twenty five patients diagnosed with mitochondrial disease caused by a mutation in mitochondrial DNA. All were contacted through a national mitochondria research clinic. The topics discussed included the experience of diagnosis, current and future health management, communication strategies and the role of reproductive technologies. The semi-structured interviews were analysed according to thematic analysis, allowing themes to emerge as the research progressed (Strauss & Corbin 1998). Because of the specific nature of mitochondrial disease, this qualitative research approach does not necessarily produce results that are generalisable to other conditions, nor can it hope to represent the experience of all those with mitochondrial disease. Instead, it produces a timely and compelling account of the potential impact of a complex, uncertain and rare genetic disorder. Ethical approval was gained through the South Wales Ethics Committee. Extracts from the interview transcripts used in this article have been anonymised to preserve patient confidentiality.

### Personal and familial experiences of illness

Through participant accounts, mitochondrial disease emerges as a highly variable genetic disorder associated with a complex network of signs and symptoms evolving over time. Pain and discomfort can become a part of normal life, and prior to diagnosis, participants were often unaware that they were experiencing what might be classified as ‘symptoms’. One man described the muscle cramps he experienced as a child, “I’ve had them all my life, it was something that wasn’t unusual and I just thought, you know, that’s part of life” [no.09]. The relatively recent discovery of mitochondrial disease meant that many respondents knew there was ‘something wrong’ long before receiving a diagnosis. One woman’s account of diagnosis highlights how the range and evolution of symptoms can be problematic for patients, families and professionals:

But to be quite honest, for years I thought there was something wrong with me and I kept going to doctors saying this is wrong, that is wrong, you know, and they’d send me for tests and I’d go back and they’d say there’s nothing wrong with you, and in the end I stopped going to doctors because I thought I was a hypochondriac. All these symptoms that I was getting, that I know now is the mitochondrial myopathy, they were poo-pooing saying oh it’s in your mind. So for years I knew that there was something wrong but then in the family, my sister was the sick one, you know, as we were younger. I looked after my sister, I wasn’t allowed to be ill because I was looking after my sister and I didn’t say anything because I thought well they’re only going to say you’re jumping on the bandwagon, you want a bit of attention as well. So you know, all these little things that were building up, I just kept them to myself and then the doctors wouldn’t help me. So it was a big relief when I found out that there was something wrong. It was a relief in one way but then in another way, when I found out, they also said that my daughter would have it, so it was a double-edged sword basically. [no.28, woman, age 59]

This single account reveals many of the complexities of the patient experience. The expansive range of symptoms associated with the disease, differing levels of severity between members of the same family and limitations of local professional knowledge compound the difficulties of diagnosis. The woman’s stance is indicative of deep familiarity with the symptoms of disease. Many participants and their families had in fact lived with the symptoms of mitochondrial disease a long time before the development of diagnostic technologies, in which case, receiving a diagnosis can be a transformative process, enabling access to a legitimate patient identity, treatment and support (Jutel 2011).

However the diagnosis of a *genetic* disease can have far reaching implications, standing in marked contrast to other types of illness because of particular associations with ‘family’ (Hallowell et al. 2003). A genetic diagnosis becomes a ‘double-edged sword’ with implications for blame and responsibility. The potential impact of ‘new biological forms of blaming’ (Nelkin & Lindee 1995) are described later. But a genetic disease should not be reduced to concern about reproductive risk. Many of those interviewed identified members of their extended family on their maternal side who were affected by disease. Most significantly, throughout their accounts, participants did not just talk about their own experiences of illness but about their observations and experiences of others.

Mitochondrial disease is a progressive, degenerative disorder. For some this trajectory was experienced as a ‘creep’, as one woman described, “it just creeps up on you and it’s something different every day” [no.15] and another described, “my health has deteriorated over the last couple of years, seen it sort of creeping up” [no.19]. Participants talked about how they were vigilant for signs of disease progression, surveilling themselves and others for signs of illness. This mapping of disease and making comparisons between oneself and others could be an emotional process, as one woman explained “it’s upsetting because *it’s like looking in a mirror*, looking at my mother and my sister, and my other sister, she’s in a wheelchair now” (no.05 emphasis added). This is a process of transgenerational observation, involving the surveillance of younger and presymptomatic members of the family. One woman described how she observed her daughter for signs of illness, specifically checking the ability to do the tasks that she was no longer able to do herself, “I keep looking, watching...my daughter can kneel down without falling over and you know different things” [no.10].

The nature of mitochondrial disease is such that symptoms and severity can differ between members of a family, which means that individuals can maintain good health while others rapidly decline. In contrast to the slow ‘creep’ of disease, for some, the implications of an uncertain illness trajectory, particularly the risk of sudden decline, appeared significant. Mitochondrial disease was described by one participant as a “waiting game” and a “time bomb” [no.26]. The uncertainty of disease progression and the implications for risk to one’s own health are compounded by the difficulties of watching a close family member suffer.

It’s difficult, I don’t know where my illness is going from one day to the next. I’ll be honest, I don’t know whether I’ll wake up in six months’ time or whether I’ve got 20 years. The problem is once you go downhill you don’t recover, you stay at the point you’re at and next time you go downhill, you get worse and eventually, 9 times out of 10, your organs start packing up. I had to sit there and watch my own brother [die] and I know it’s a possibility that may be me as well. [no.18, man, age 42]

This man’s account reveals the impact of diverse illness trajectories. Whereas mitochondrial disease is generally progressive, the path ‘downhill’ can be unpredictable and impossible to map. So for this man, the fact that “you never know what’s going to happen next” [no.18] was compounded by his experience of watching his brother fall seriously ill. The clues that he uses to inform his own future are his observations of watching the decline of a close member of the family. These techniques of self and mutual surveillance (Featherstone et al. 2006) are key for understanding how patients and family members position themselves and others, and how futures are projected. During the interviews, participants did not necessarily talk about the technical aspects of the causes and genetic complexities of mitochondrial disease. Instead many displayed a deep and extensive knowledge of its consequences on their own and others’ bodies, lives and social relationships. This wealth of experiential knowledge, gained through experience and observation, often involving several generations, highlights how the disease can become both normalised and feared through every day practices (Forrest et al. 2008).

#### Asymptomatic patients and the generation gap

There is no doubt that mitochondrial disease can be devastating and distressing. Many participants who experienced serious symptoms or who had watched the sudden decline of family members gave thick descriptions of living with mitochondrial disease, describing it as “a death sentence” [no.03], “a wretched disease” [no.02], “a horrible thing to have” [no.18] and “hard to live with” [no.07]. These accounts are informed by the personal and collective experiences of illness and deterioration. However, not all patients respond to mitochondrial disease in this way. The finding that differences exist along generational lines is critical when thinking about the target population for reproductive technologies.

Genetic technologies have the potential to create new patient populations by separating the diagnostic process from experiential awareness of symptoms. ‘Patients without symptoms’ (Finkler et al. 2003) include those ‘at risk’ of developing a late onset condition (in the case of Huntington disease for example) and carriers of a genetic disorder (in the case of recessive disorders such as cystic fibrosis or X-linked disorders such as haemophilia). Mitochondrial disease fits both these categories of patient-hood in which case an individual with or without symptoms might not consider themselves to be a ‘patient’. Through participant accounts, a picture emerges of a younger generation of adults with experiences very different from those of their parents or grandparents. This is not surprising given the late onset degenerative nature of the disease. However, highlighting the experiences of disease and identifying differences according to generation sheds light on potentially diverse responses to reproductive risk.

Overwhelmingly, for most participants the *diagnosis* of mitochondrial disease did not appear to be problematic. One reason for this appears to be the normalisation of symptoms and illness experiences. Mitochondrial disease is associated with an extensive range of symptoms, some of which appear in childhood and become accepted as ‘normal’. All the participants were adults when they received a diagnosis. In addition, the normalisation of illness appears to be facilitated when the illness is known within the family. Many participants were diagnosed at the same time as others in the family, or were aware that others in the family had already been diagnosed. Diagnosis therefore does not necessarily mark the beginning of the patient journey nor lead to biographical disruption (Bury 1982). This was the case for one woman who had received her diagnosis when she was nineteen years old, reflecting, “I knew there was this thing within the family and it was making the people poorly, and it was genetic, but that was it, I didn’t really take much interest in it” [no.16]. Readiness to receive and assimilate health information is dependent on many factors including age and life stage (Gregory et al. 2007). Thus for a generation of adults who might be relatively asymptomatic, and who are not currently thinking about their reproductive future, the diagnosis of a genetic disease does not necessarily trigger the search for technological intervention.

Whereas normalisation of symptoms and experiences might reduce the impact of diagnosis for some, others identified how the nature of the disease led to disengagement. A serious and life limiting disorder with limited treatment options is a potent combination and several participants described how their adult children did not want to attend the specialist mitochondrial disease clinic or seek medical attention.

Because I keep saying to my sons, do you want to go and have the test for it? And they say, well, what’s the point if there’s no cure? What can we do about it? There isn’t a tablet that you can say, take this and it’ll make you better. [no.14, woman, age 56].

In describing her adult sons’ reluctance to seek confirmation of their own health status or risk to health, this mother identifies the tension inherent within genetic medicine, the separation of biomedical knowledge from experience. Mitochondrial disease is unusual as a genetic disease because of the complex pattern of mitochondrial inheritance. All the offspring of a woman with mitochondrial disease will inherit a degree of defective mitochondria but a blood test or muscle biopsy might identify the specific nature and extent of mitochondrial dysfunction. Compared to single gene disorders, this challenges the boundaries between health, illness and being ‘at risk’. However, with the dearth of research exploring patient responses to mitochondrial disease, useful comparisons can be drawn with other late onset conditions such as Huntington’s Disease, where the profound social and emotional effects are better explored (Richards 2004) and where the uptake of presymptomatic testing is low (Evers-Kiebooms et al. 2002). The availability of treatment for disease is an important consideration in understanding the ways in which individuals respond to genetic knowledge. The mother’s words suggesting her persistence in encouraging her sons to be tested, highlight a potential tension between current and future health needs, the right ‘not to know’ and the range of actors with investment in these decisions. In recognition of the limitations of current genetic knowledge, some participants expressed a strong desire to offer protection to their adult children.

[Son] did come to an information event with us, only one, he hasn’t been back. […] He’ll make a point of saying I don’t want it and I don’t want to talk about it […] He’s young, he’s fit, he wants to get on with his life. And I don’t take that away from him and his cousins because to me that’s what they need to do because looking back all I can remember was my mother being bad, and then seeing my brothers and sisters, and you think, well this is all I ever know is someone being bad in the family. [no.26, woman age 52]

Many participants recognised why their children might not want to identify with an illness identity, sanctioning the decision to ‘get on with life’. The protection afforded to offspring is not just about shielding them from personal experience of illness but it also involves protecting this generation from the family experience of illness. For this mother it was important to know that her son, nieces and nephews would not share the experience of caring for others. We know that women are the key communicators of genetic knowledge within the family (Richards 1996), even undergoing genetic testing to provide risk information for others (d’Agincourt-Canning 2001). The gendering of roles and responsibilities was in evidence in this study, potentially accentuated by the maternal inheritance pattern of mitochondrial disease. When talking about their younger relatives, many of the female participants performed the role of ‘kin keeper’ (Young & Willmott 1957) by encouraging their adult children, nieces and nephews to prioritise their health. Some participants expressed concern that the disease was not being taken seriously. One woman for example mentioned “[Niece] thinks we’re all barking mad, we’re looking to be ill and all this, even though she suffers a lot herself with different problems” [no.07]. Whereas the rejection of an illness identity can be cast as a choice in the context of a disease with limited options for treatment, this was not always the case. For one mother, the explanation as to why her nephews and nieces refused to attend clinic was “they reckon ignorance is bliss” [no.15]. Awareness of the progressive nature of the disease was also evident, as one parent warned, “little do they know it might be them in ten or twenty years time” [no.03].

Responses to diagnosis of disease or risk are informed by personal stocks of knowledge (Parsons & Atkinson 1992; Michie & Marteau 1996). The reasons why individuals choose to distance themselves from the disease and specialist management are multiple, including the relevance of health and genetic information, ‘getting on with life’ and ‘ignorance is bliss’. However, these strategies might have serious implications. In the following extract a mother describes taking her two year old daughter to hospital following a bout ‘stiffness’, demonstrating considerable barriers to communication about mitochondrial disease:

Interviewee: They look at you stupid in the hospital and you think well I’m not bringing my daughter in here for no reason

Researcher: So did you mention mitochondria then?

Interviewee: No. Should I have? […] Do you know I have mentioned it in the past and I think because of the blankness I get and it doesn’t seem to, they don’t care anyway. I could have mentioned it and it wouldn’t have made any difference. My mother says to me, like when they put me on these tablets, did you mention mitochondria? And I said well they know, I’ve mentioned it before and she said yes but did you mention it again? And I think, well I’ve told them before and they didn’t really give a stuff then so I don’t see any point in telling them again. [no.25, woman, age 24]

This respondent was diagnosed with mitochondrial disease alongside her mother, aunts and sisters and during the interview she talked about fatigue and the difficulties of a physically demanding job. This woman’s mother and aunt were interviewed and the impact of the disease was also prominent in their accounts. Yet for this woman, neither her own diagnosis of mitochondrial disease nor the child’s potential diagnosis (the daughter is at risk due to the nature of maternal inheritance) was used as a resource to explain the signs of illness or to communicate potential explanations to hospital staff. This example brings into sharp relief the ways in which knowledge of mitochondrial disease might be translated by following generations, emphasising the challenges that rare and complex disease presents for patients and health services.

Through describing their own experiences, and telling stories about sons, daughters, nieces and nephews, this research has revealed a generation of adults with mitochondrial disease who might be described as a hard to reach group. The tension between disengaging or being shielded from the personal and collective experience of illness and concern for current health suggests a key role for counsellors and clinicians in identifying transgenerational communication patterns concerning health, illness and genetic risk.

#### Reproductive technologies and the family

A genetic diagnosis has been described as a ‘double edged sword’ where “[genetic] explanations have a double edge, for while they shift responsibility to DNA, they create a new biological form of blaming – for the ‘flawed’ parents who pass on ‘bad genes’ , for those who knowingly take genetic risks (Nelkin & Lindee 1995). The development of techniques of mitochondria donation presents a pertinent opportunity to examine the implications of diagnosis and reproductive risk for patients with mitochondrial disease.

The aetiology of mitochondrial disease is complex and clinical consequences vary greatly, even between mother and child (Poulton et al. 2003). For many types of mitochondrial disease, a ‘grey zone’ exists where the risk of developing symptoms, the type of symptoms and severity are difficult to estimate (Bredenoord et al. 2009) leading to difficulties in assessing reproductive risk and predicting outcome (Bredenoord et al. 2010). How patients and those ‘at risk’ approach reproductive technologies has been explored in multiple contexts, including single gene disorders, late onset disorders and more complex disorders such as cancer (Decruyenaere et al. 2007; Myring et al. 2011; Ormondroyd et al. 2012). In contrast, there has been little reflection about the contexts within which reproductive decisions might be made by those with mitochondrial disease.

Technologies such as pre-implantation genetic diagnosis and prenatal diagnosis which are currently available offer women with mitochondrial disease the opportunity to reduce their risk of having a child with the disease (Poulton et al. 2010). Few participants in this study had experience of using assisted reproductive technologies, reflecting the low uptake of these technologies at the national level (Poulton & Oakeshott 2012). In the context of limited research in this vital area, this final section presents a valuable case study of one woman’s experience of using assisted reproductive technology. This woman’s decision to use prenatal diagnostic technologies was triggered by the sudden illness of a close family member. Specifically, this extract points to the woman’s response and subsequent outcome following prenatal diagnosis:

It was a baby boy, but he had 68% mutation and my mother had a similar percentage so I was able to compare what could happen to my baby. We then made the decision [to abort] because I could see what my mother was like and also my wider family […] We would be bringing a baby into the world knowing that at some point it wasn’t going to have its health so we walked away and we thought, as much as we don’t want to do this, it is the right thing to do. It was not an easy decision to make, and we discussed this with our whole family, everyone who is affected by this mutation […] Our final decision came down to me and my husband saying if our eldest son ever says, well why did you not do anything with me, why did you bring me into the world - simple because we didn’t know. We didn’t understand and we didn’t realise what could happen. But with that baby, we couldn’t have done that. It would have been purely selfish reasons as to have just said, well no actually we’re still going to let him come into the world. [no.16, woman, age 33]

How individuals make sense of uncertainty and complexity is a pertinent question in the context of reproductive options. This is a highly personal account of making decisions on basis of prenatal diagnostic information, specifically the identification of a mutation level of 68%. Recognition of multiple interpretations of biomedical information have a long history, particularly in the translation of statistical risk (Gillespie 2012). Risk analysis relies on techniques of classification, presenting possibly reductionist diagnostic categories which have the capacity to challenge and reconfigure existing boundaries between health and illness. Genetic diagnosis continues to involve complex levels of clinical decision making, even where a genetic mutation has been identified (Hedgecoe 2003; Miller et al. 2005; Whitmarsh et al. 2007). Mitochondrial disease as a highly complex disease classification falls into this category, where the presence of mutated mitochondria does not necessarily lead to a diagnosis of mitochondrial disease.

This single account is important because it reveals how biomedical information and the diagnostic criteria of mutation ratio, can be interpreted. Whereas mutation ratio is used as a signifier to measure and project severity of disease, this can only be made meaningful in the context of concrete experience of the disease. For this woman, this process involved identifying the closest match within the family and then making a decision based on that family member’s current health. This account therefore highlights the centrality of experiential (personal and familial) knowledge in reproductive decision making. The matching of genotype (of the baby) with phenotype (of the living adult) was possible because the mutation ratio of individuals in this family were known. But this reflects a key issue within genetic medicine which is the ownership and disclosure of personal and collective information. Should this information about the health status of family members, which appears to be significant in informing the decision making process, be disclosed if the potential parents do not already know? In addition, assessment of reproductive risk allows informed reproductive choice (Ten Kate 2012) but it also holds the potential to reveal unrequested information, including the woman’s own mutation level, in which case concerns might be raised about presymptomatic testing (Skirton et al. 2013).

Finally, and returning to the development of novel reproductive techniques, the evidence submitted to the Nuffield Council on Bioethics (Nuffield Council on Bioethics 2012) suggests widespread support from patients and professionals. However it is clear that we have a lot more to discover about the use of technologies in practice. Vital questions remain about how mitochondrial disease is experienced, measured and communicated, including how mutation ratio combines with experience to provide estimations of risk and projections of the future, how normative decisions are made about acceptable levels of mitochondrial mutation and how the boundaries between normal and pathological are negotiated by both patients, families and health professionals.

## Conclusion

Mitochondrial medicine represents a rapidly changing field with newly emerging tools for diagnosis and risk assessment. This article raises questions about responses to risk, communication of health information and the role of technological solutions in the context of complex and uncertain biomedical knowledge. The extensive interest in mitochondria donation shown by policy-makers, interest groups and the media highlight the significant challenge to existing legal and ethical frameworks. It also reveals a gap between the capabilities of health technologies and knowledge of the practical realities of living with genetic disease. Except for a few key exceptions (Noorda et al. 2007; Bredenoord et al. 2011) the implications of introducing new technologies into the clinic have been missing from the debate. This article has paid attention to the accounts of adults with mitochondrial disease, and builds on the limited literature addressing the perspectives of patients and families. Primarily, the modest research that has been conducted has focused on the parental experience, characterised as extremely stressful (Kim et al. 2010; Read 2003) and one study conducted with a large family cohort of adult patients identified the relationship between maternal inheritance, blame and responsibility (Featherstone et al. 2006).

The identification of a patient population who do not choose to attend specialist clinics is an important finding. Clinical contact with those at risk is considered important for early intervention for long term health (Schaefer et al. 2008) but it is also important to ensure patient choice and activity in the promotion of informed decision making (Decruyenaere et al. 2007). In combination with the maternal inheritance pattern of mitochondrial disease, this disease clearly lays different burdens on women than it does on their male counterparts. The development of reproductive technologies highlights the gendered impact of disease and its technologies (Chadwick 2009), potentially leading to surveillance of sexuality and lifestyle of young women. Finally, genetic technology informs a new concept of ‘responsible’ parentage where prior knowledge of risk appears significant when negotiating responsibility and the potential for blame (Finkler 2000). How patients and potential parents account for their use and non-use of reproductive technologies will be a vital question for future research.
